# Workers Memorial Day — April 28, 2017

**DOI:** 10.15585/mmwr.mm6616a1

**Published:** 2017-04-28

Workers Memorial Day, observed annually on April 28,* recognizes workers who suffered or died because of exposures to hazards at work. In 2015, work-related injuries claimed the lives of 4,836 U.S. workers.^†^ Although deaths resulting from work-related injuries are captured by surveillance systems, most deaths resulting from work-related illness are not. In 2007, an estimated 53,445 persons died from work-related illness (*1*). In 2015, employers reported approximately 2.9 million nonfatal injuries and illnesses to private industry workers and 752,600 to state and local government workers^§^; an emergency department surveillance system estimates that 2.7 million workers were treated for work-related injuries in emergency departments, resulting in 85,000 hospitalizations (National Institute for Occupational Safety and Health [CDC-NIOSH], unpublished data, 2017).

Occupational injuries and illnesses also have economic costs. The societal cost of work-related fatalities, injuries, and illnesses was estimated at $250 billion in 2007, based on methods that focus on medical costs and productivity losses (*1*).

New data on fatal falls in the oil and gas industry are reported in this issue of *MMWR*. CDC-NIOSH collects detailed information about these fatal events in its Fatalities in Oil and Gas Extraction Industry database (https://www.cdc.gov/niosh/topics/fog/about.html) and uses these data to inform the industry and other stakeholders about health and safety risks and to guide intervention activities.

## Footnotes

* Workers Memorial Day was established in 1970 by the American Federation of Labor and Congress of Industrial Organizations.

† National Census of Fatal Occupational Injuries in 2015. https://www.bls.gov/news.release/pdf/cfoi.pdf.

§ Employer-reported workplace injuries and illnesses in 2015. https://www.bls.gov/news.release/pdf/osh.pdf.
